# The Effect of shape on Cellular Uptake of Gold Nanoparticles in the forms of Stars, Rods, and Triangles

**DOI:** 10.1038/s41598-017-04229-z

**Published:** 2017-06-19

**Authors:** Xueping Xie, Jinfeng Liao, Xiaoru Shao, Qianshun Li, Yunfeng Lin

**Affiliations:** State Key Laboratory of Oral Diseases, National Clinical Research Center for Oral Diseases, West China Hospital of Stomatology, Sichuan University, Chengdu, 610041 P. R. China

## Abstract

Gold nanomaterials have attracted considerable interest as vehicles for intracellular drug delivery. In our study, we synthesized three different shapes of methylpolyethylene glycol coated-anisotropic gold nanoparticles: stars, rods, and triangles. The cellular internalization of these nanoparticles by RAW264.7 cells was analyzed, providing a parametric evaluation of the effect of shape. The efficiency of cellular uptake of the gold nanoparticles was found to rank in the following order from lowest to highest: stars, rods, and triangles. The possible mechanisms of cellular uptake for the three types of gold nanoparticles were examined, and it was found that different shapes tended to use the various endocytosis pathways in different proportions. Our study, which has demonstrated that shape can modulate the uptake of nanoparticles into RAW264.7 cells and that triangles were the shape with the most efficient cellular uptake, provides useful guidance toward the design of nanomaterials for drug delivery.

## Introduction

Gold nanoparticles (GNPs), which have unique properties, have been attracting increasing attention in the fields of drug and gene delivery^1–3^, medical imaging^4^, and cancer treatment^5, 6^. GNPs have numerous advantages for biomedical applications, including the ease of adding functional biomolecules, efficiency in penetrating cells, and their ability to respond to light in near-infrared^7–9^. However, a better understanding is needed of the interaction of GNPs with biological membranes. The size, shape, surface charge, and surface coating of nanoparticles all can affect their interactions with cells^10^. Chan and coworkers demonstrated that the cellular uptake of GNPs was strongly size-dependent, with 50 nm nanoparticles showing the highest uptake by HeLa cells among a set of GNPs that ranged from 10 nm to 100 nm^11^. Surface charge can also have an effect on cellular uptake. It has been shown that electronegative particles exhibited a lower efficiency of cellular uptake compared to electropositive nanoparticles, as assessed using monocyte-derived dendritic cells^12^. Saha *et al*. demonstrated that the surface coating of gold nanoparticles had a significant influence on the endocytosis mechanisms used by HeLa cells and MCF10A cells^13^.

Anisotropic GNPs have shape-dependent physical and chemical properties^14^. In recent decades, various gold nanostructures have been produced, including triangles^15^, stars^16^, cubes^17, 18^, octahedrons^19^, plates, and prisms^20, 21^. A greater understanding of the shape effect on GNP-cell interactions would aid the development of effective tools for drug delivery. However, there have been few studies on cellular uptake of GNPs with different shapes and most of the attention has been given to spherical nanoparticles. It has been shown that the cellular uptake of rod-like GNPs by HeLa cells is less efficient than that of spherical ones^12^. Cho *et al*. chose gold nanospheres and gold nanocages to investigate the effects of shape, size, and surface functional group on cellular uptake^22^. Their results suggested that shape did have an influence, but the GNPs with different shapes also had different sizes and surface functional groups leading to ambiguity in how much of the observed effects were due to shape alone. Recently, Nambara *et al*. suggested that the triangular gold nanoparticles showed more effective cellular uptake than did spherical ones with similar surface area and this difference was more obvious in HeLa cells than that in RAW264.7 cells^23^.

**Figure 1:**
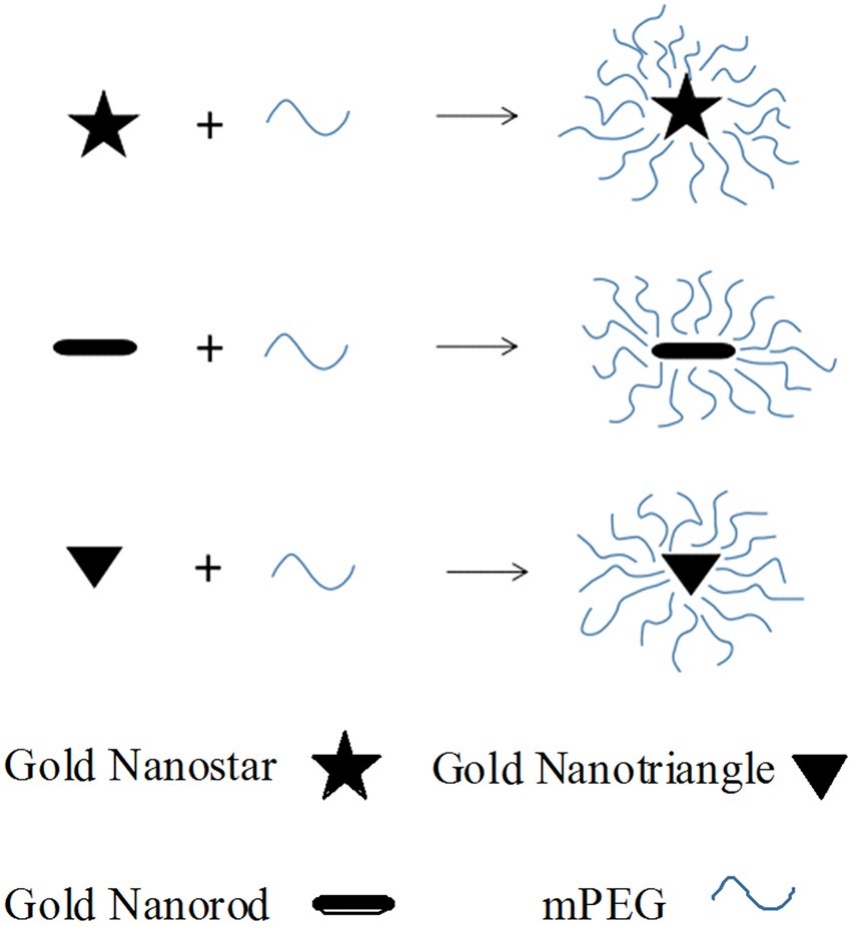
Schematics of gold nanostar, gold nanorod and gold nanotriangle loading into mPEG.

In our study, we chose three anisotropic geometries, star, rod, and triangle, to investigate the shape effect on cellular uptake into RAW264.7 cells. We fabricated gold nanostars (GNSs), gold nanorods (GNRs) and gold nanotriangles (GNTs) with similar size and coated them with methylpolyethylene glycol (mPEG) to obtain a neutral surface charge for excluding the interference of other factors (Fig. 1). As is typically observed, mPEGylation allowed the GNPs to be well dispersed in aqueous solution and reduced the toxicity of the stabilizing agent CTAB^24, 25^. The mPEG outer layer of mPEG coated gold nanoparticles (P-GNPs) can effectively prevent the adhesion of plasma proteins and subsequent phagocytosis by the immune system; this improves the circulation time of GNPs^26, 27^. For our research, we used RAW264.7 cells, which are mouse leukaemic monocyte macrophage^24^. Different from commonly used cancerous cells such as HeLa cells and breast cancer cells^11, 12, 23, 28–31^, RAW264.7 might be another cell model for the study of cellular uptake of GNPs. Arnida *et al*. and Nambara *et al*. did evaluate the cellular uptake of GNPs by RAW264.7, but they did not investigate the endocytosis pathways^24, 26^. In this article, we report on our investigations into the cellular uptake of three types of GNPs by RAW264.7 and discuss the possible endocytosis mechanisms.

## Results

### The Characterization of GNPs and P-GNP*s*

**Figure 2:**
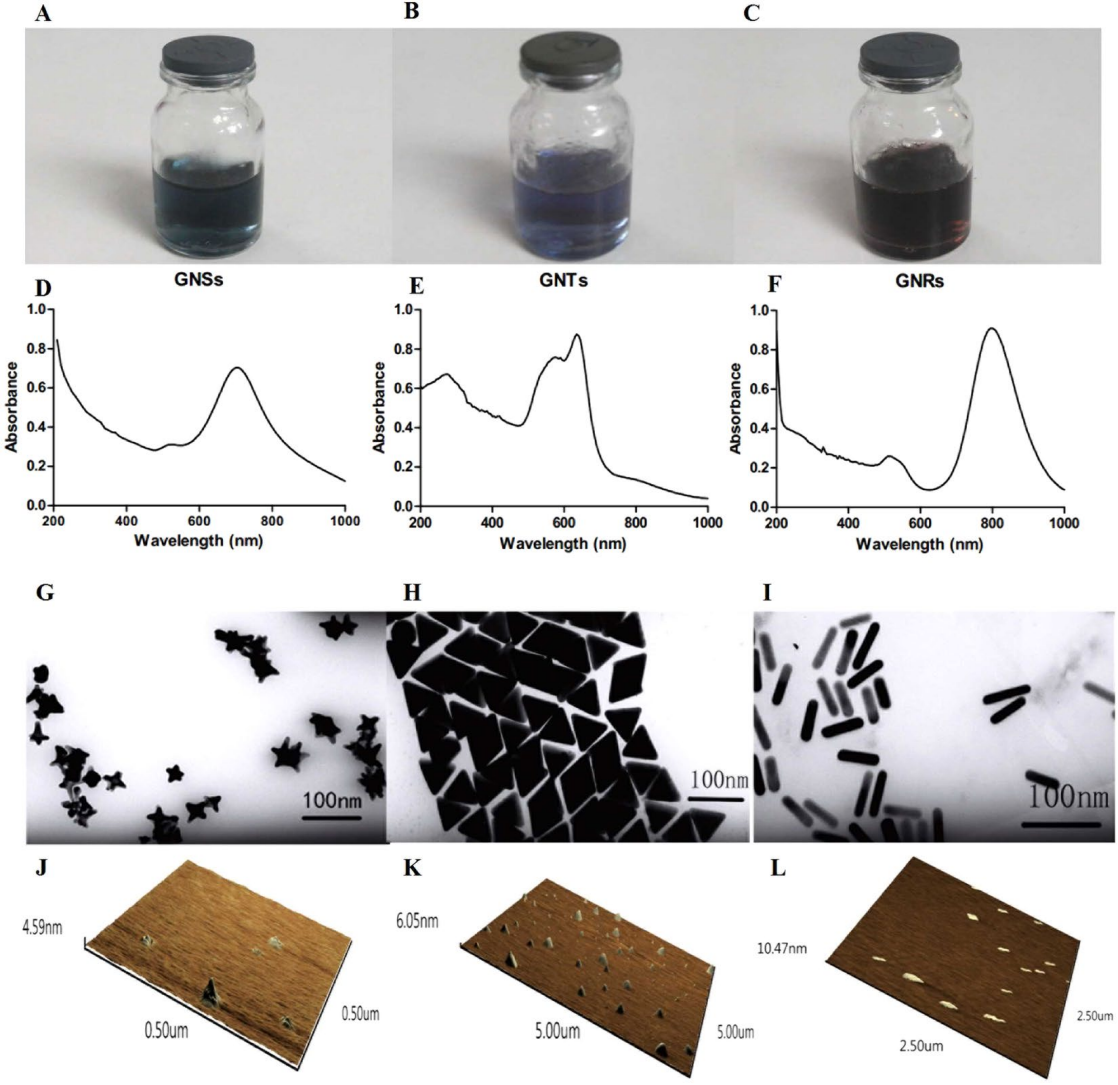
Photographs, UV-Vis spectra, TEM and 3D AFM images of GNSs (**A**,**D**,**G**,**J**), GNTs (**B**,**E**,**H**,**K**) and GNRs (**C**,**F**,**I**,**L**).

The visible colors and optical properties of GNPs are well-known to be extremely sensitive to their shape and size^32^. As shown in Fig. 2A–C, the GNPs exhibited distinct color variations. The color of GNSs, GNTs, and GNRs is in turn dark green, blue, and wine red. The optical properties of GNPs were characterized by UV-Vis spectroscopy. The two surface plasmon resonance (SPR) peaks of GNSs were observed at 525 nm and 705 nm (Fig. 2D), which corresponded to the transverse longitudinal plasmon resonance of the elongated tips. The GNRs were observed to have two SPR peaks at 510 nm and 800 nm, which were also related to the transverse and longitudinal modes (Fig. 2E). As shown in Fig. 2F, the GNTs had a major plasmon band at 635 nm corresponding to the in-plane band; the band at 575 nm was related to the byproducts of the gold nanoparticles. The morphologies of the GNPs were characterized by TEM (Fig. 2G–I). From the TEM images, the GNSs, GNTs, and GNRs had star, triangle, and rod-like structures matching their designs. The mean size of GNPs was ~50 nm and the GNPs were monodispersed. The UV-Vis spectra corresponded to the TEM images. As a complementary characterization tool, AFM was used to determine the 3D structure of the GNPs (Fig. 2J–L).

**Figure 3:**
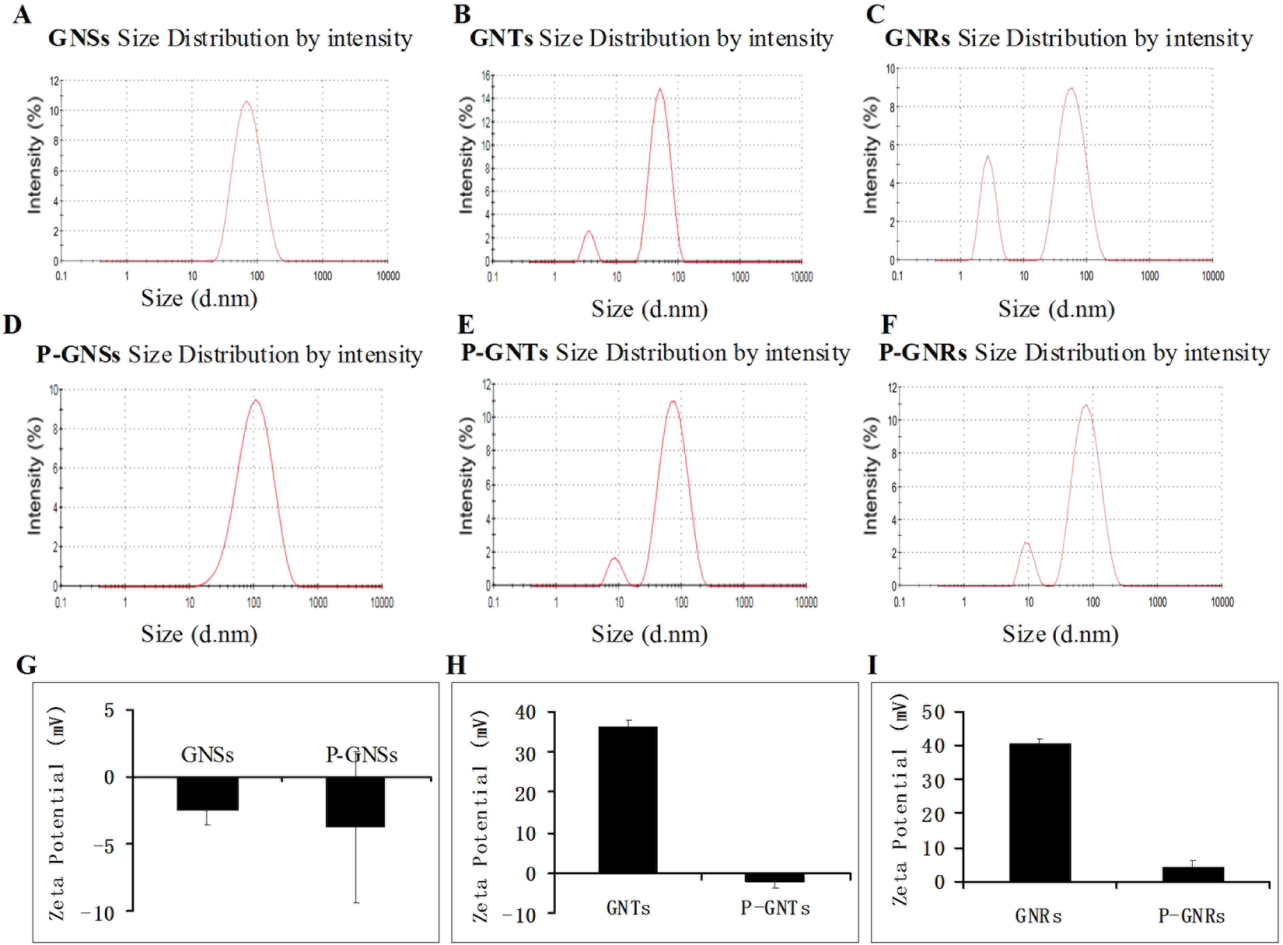
Size and zeta potential of GNPs and P-GNPs: Size of GNSs, GNTs, GNRs(**A**,**B**,**C**), Size of P-GNSs, P-GNTs, P-GNRs(**D**,**E**,**F**), Zeta potential of GNSs and P-GNSs(**G**), GNTs and P-GNTs (**H**), GNRs and P-GNRs (**I**).

The hydrodynamic sizes and zeta potentials of GNPs (Fig. 3A–C) and P-GNPs (Fig. 3D–F) dispersed in water were measured by dynamic light scattering (DLS). The hydrodynamic size of GNPs was ~60–70 nm. After mPEG coating, the hydrodynamic size increased from ~60–70 nm to ~80–90 nm, which is in line with expectations. GNSs had neutral surface charge, whereas GNTs and GNRs were highly positively charged. They all possessed a neutral potential after modification with mPEG (Fig. 3G–I). Then we obtained the nanoparticles with similar sizes and surface potentials, as shown in Table 1. The shape of the GNPs become the major variable parameter for our cellular uptake research.

**Table 1:** Hydrodynamic size, zeta potential and polydispersity index (PDI) of various GNPs. Data are provided as mean ± S.D. (n = 3).

Sample	Size (nm)	Zeta potential (mV)	PDI
GNSs	68.55 ± 5.28	−2.47 ± 1.16	0.27 ± 0.05
GNTs	61.33 ± 1.64	36.07 ± 1.60	0.24 ± 0.10
GNRs	70.49 ± 10.05	40.53 ± 3.93	0.31 ± 0.02
P-GNSs	82.88 ± 3.29	−3.87 ± 5.58	0.29 ± 0.04
P-GNTs	84.57 ± 2.67	−1.88 ± 1.77	0.25 ± 0.01
P-GNRs	90.81 ± 3.15	1.14 ± 2.35	0.33 ± 0.07

### *In Vitro* Cytotoxicity of P-GNPs

**Figure 4:**
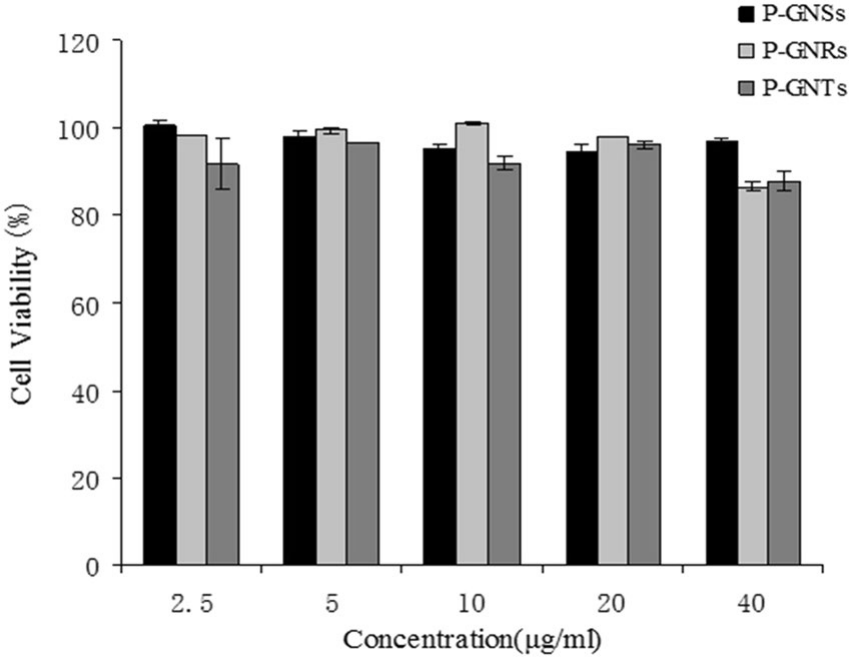
Relative viabilities of RAW 264.7 after being incubated with various concentrations of P-GNPs for 24 h, as determined using CCK8 assays. Data represent mean ± SEM (n = 3).

To select a safe concentration of P-GNPs for the cellular uptake studies, cytotoxicity was evaluated using the CCK-8 assay. Treatment of cells with P-GNPs for 24 h revealed that these nanoparticles were nontoxic over the concentration range of 2.5 μg/mL to 40 μg/mL. Differences in cytotoxicity among these three were not significant (Fig. 4). We chose 20 μg/mL as a safe concentration for studying cellular uptake.

### Cellular Uptake of P-GNPs

**Figure 5:**
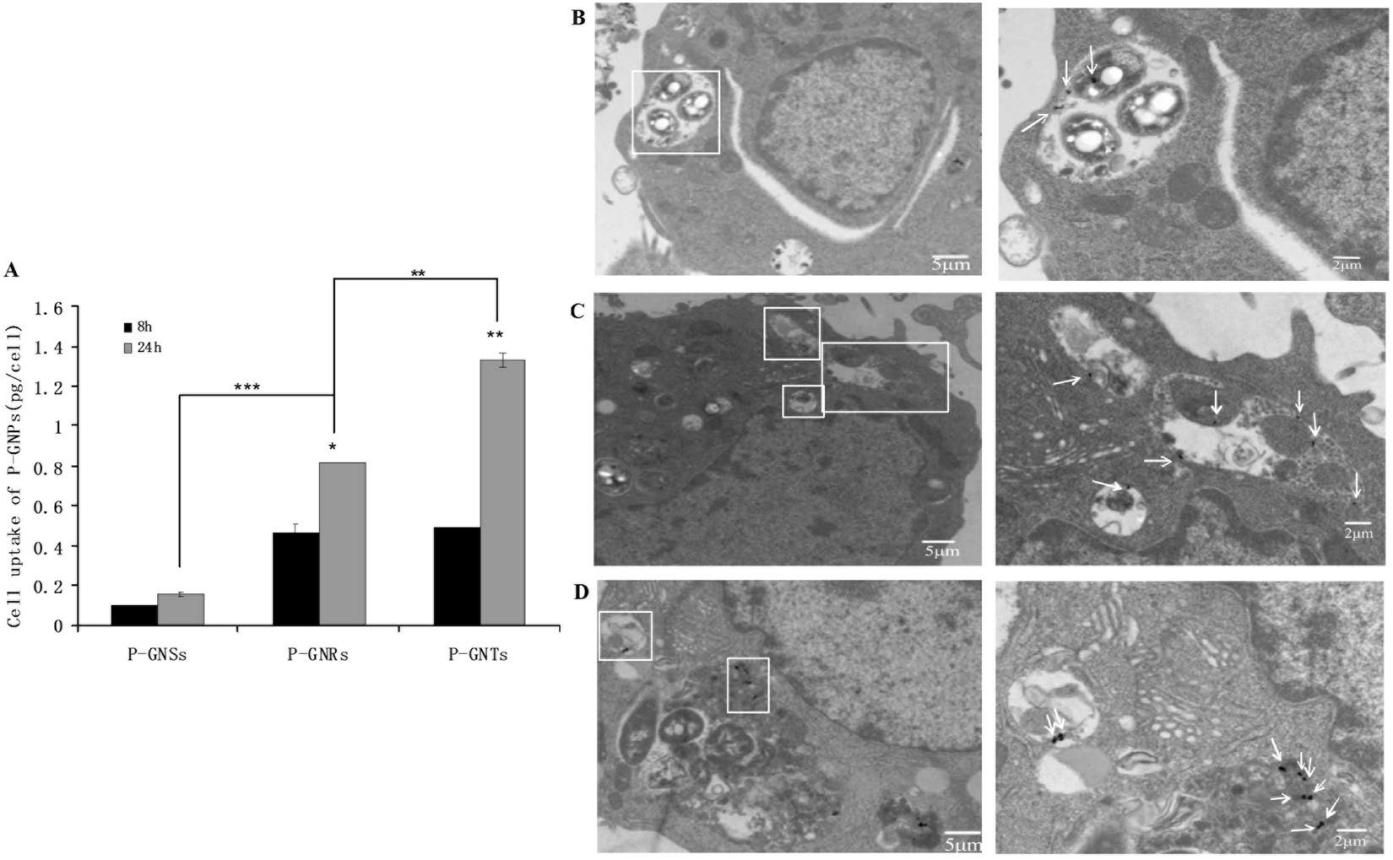
Cellular uptake of P-GNPs(**A**) and TEM images of RAW 264.7 after 24 h of incubation with P-GNSs(**B**), P-GNRs(**C**), P-GNTs(**D**). Data represent mean ± SEM (n = 3). Statistical significance is represented by *p < 0.05, **p < 0.01, ***p < 0.001.

The intracellular concentrations of gold after incubation for 4 h with the P-GNPs were below the detection limit of inductively coupled plasma atomic emission spectrometer (ICP-AES). At 8 h and 24 h, shape and time-dependent cellular uptake was observed (Fig. 5A). After incubation for 24 h, gold concentrations in the cells incubated with P-GNSs, P-GNRs, and P-GNTs were 0.154 ± 0.010 pg/cell, 0.814 ± 0.001 pg/cell, and 1.333 ± 0.038 pg/cell, respectively. When the data were converted to percentage uptake from the total added gold the results were 0.38%, 2.04%, and 3.33%. The cellular uptake of P-GNTs was the significantly greatest, followed by P-GNRs and P-GNSs. The intracellular concentrations of gold after incubation for 8 h with P-GNSs, P-GNRs, and P-GNTs were 0.098 ± 0.0003 pg/cell, 0.463 ± 0.047 pg/cell, and 0.488 ± 0.003 pg/cell, respectively. In term of % added, the uptake from the total gold were 0.25%, 1.16%, and 1.22%. The cellular uptake of P-GNPs was higher at 24 h than at 8 h. The results suggested that nanoparticle shape played an important role in cellular uptake. TEM images of cellular uptake and localization of P-GNPs showed that nanoparticles were internalized as single particles after 24 h incubation (Fig. 5B–D) and were localized in vacuoles (i.e., endosomes and/or lysosomes) in the perinuclear region of the cells. Nanoparticles were not found in the nucleus.

### The Cellular Uptake Mechanisms of P-GNPs of Different Shapes

**Figure 6:**
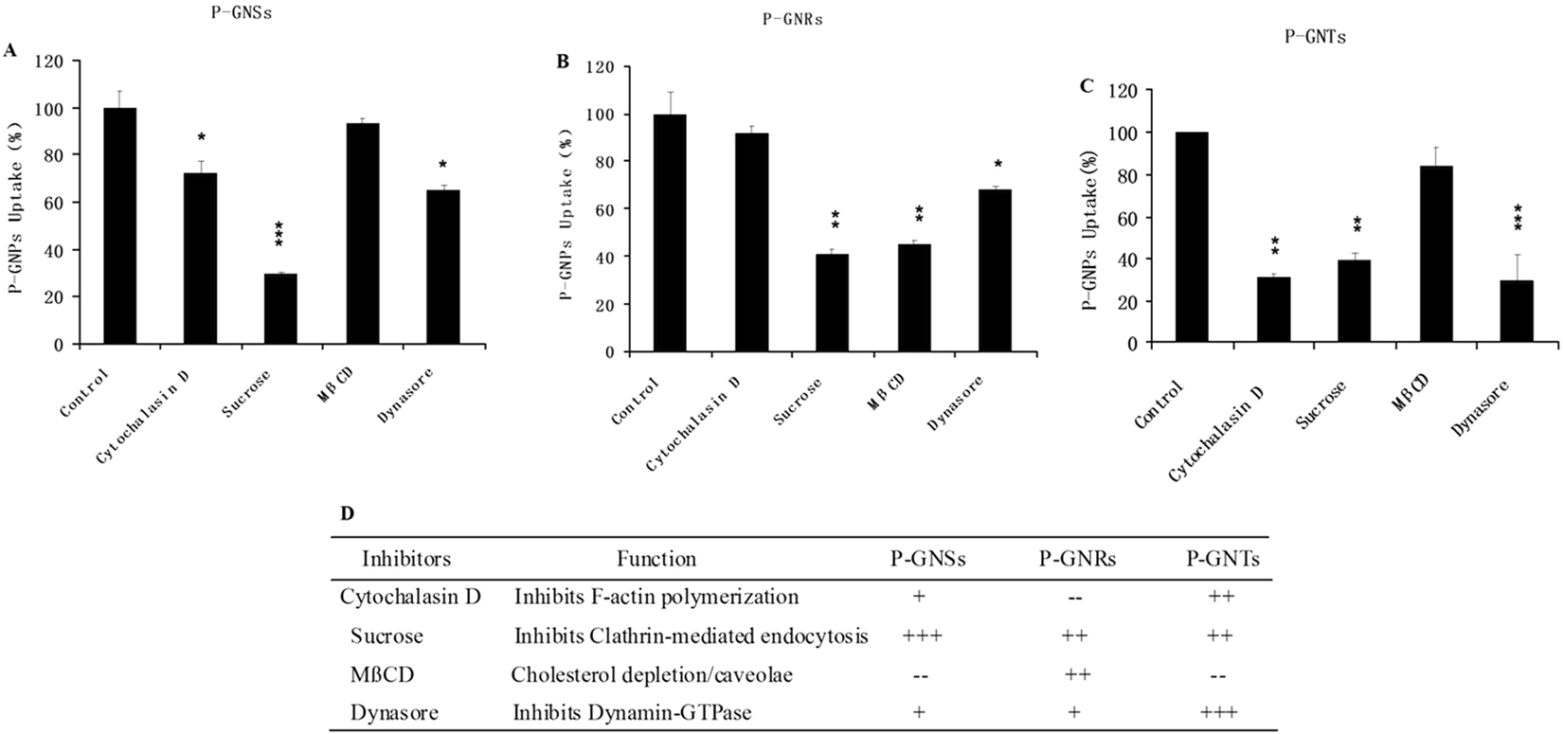
Uptake % of P-GNSs (**A**), P-GNRs (**B**) and P-GNTs (**C**) (compared to the positive controls) in the present of different endocytic inhibitors in the RAW264.7 cells. Error bars represent standard deviation. *p < 0.05, **p < 0.01, ***p < 0.001 compared to the control. (**D**)Summary of cellular uptake inhibition of P-GNPs in the present of endocytic inhibitors. +p < 0.05; ++p < 0.01; +++p < 0.001; – no significant inhibition.

We used endocytic inhibitors to evaluate the involvement of different endocytic pathways in the uptake of these three types of gold nanoparticles. Membrane invagination during micropinocytosis requires actin filament reorganization^14, 33^. To investigate the effect of cytoskeletal rearrangement on nanoparticle uptake, we utilized cytochalasin D to disrupt F-actin polymerization. The uptake of P-GNSs and P-GNRs showed weak inhibition, but P-GNTs showed 69% ± 1.66% inhibition relative to the control. Additionally, the pretreatment of cells with sucrose, an inhibitor of clathrin-mediated endocytosis, dramatically reduced the uptake of all three particle types, demonstrating that they all could be internalized into RAW264.7 cells via clathrin-mediated pathways. Next, we studied the effect on uptake of MβCD, a cholesterol depletion agent that inhibits caveolae/lipid raft-mediated endocytosis. Strong uptake inhibition (55% ± 1.65%) was only observed for P-GNRs. Finally, we pretreated cells with Dynasore, an effective inhibitor of dynamin-dependent endocytosis. We found that Dynasore pretreatment significantly inhibited the internalization of P-GNTs (71% ± 12.19%) (Fig. 6A–C). Taken together, the data support the conclusions that P-GNSs are prone to enter cells through clathrin-mediated uptake, and P-GNRs are internalized into cells through both clathrin- and caveolae/lipid raft-mediated endocytosis. The P-GNTs showed multiple endocytosis pathways, including clathrin-mediated endocytosis and a dynamin-dependent pathway. In addition, cytoskeletal rearrangement is strongly related to the uptake of P-GNTs (Fig. 6D).

## Discussion

Three anisotropic gold nanoparticles: stars, rods, and triangles were synthesized and coated with mPEG and used to investigate the effect of shape on cellular uptake. The mean size of GNPs measured by TEM was ~50 nm and the hydrodynamic size of GNPs was ~60–70 nm. Samples used for DLS were in aqueous solution, whereas samples were dried at room temperature for TEM analysis. Due to the layer of hydration around GNPs in aqueous solution, DLS measurements were bigger than the diameters shown in TEM measurements^34^. After mPEG modification, the hydrodynamic size increased from ~60–70 nm into ~80–90 nm. Since the seedless synthesis method of GNSs was different from the seed-mediated method of GNTs and GNRs, GNSs stabilized in a HEPES solution had neutral surface charge, whereas GNTs and GNRs stabilized in CTAC and CTAB solutions were highly positively charged. After mPEG modification, they all changed into neutral surface charge. The changes of hydrodynamic sizes and zeta potentials indicated that mPEG was successfully coated onto the surfaces of GNPs. Therefore, in our study we obtained there different shaped nanoparticles with similar sizes and surface potentials.

The cellular internalization of P-GNPs was studied by exposing the nanoparticles to RAW264.7 cells, followed by extensive washing to remove nanoparticles adsorbed to the cell surface. 20 μg/ml of P-GNPs were used as a safe concentration for cellular uptake study determined by CCK8 assay. The uptake of P-GNPs was quantified using ICP-AES. We found the cellular uptake of P-GNPs were increased in the order of P-GNSs, P-GNRs and P-GNTs. What, more, after incubation for 24 h, the cellular uptake of P-GNPs were higher than that for 8 h which showed time- dependent cellular uptake. The results suggested that nanoparticle shape played an important role in cellular uptake.

We tried to elucidate the mechanisms leading to the observation of different preferred modes of cellular uptake. Particles can be internalized into cells through two major mechanisms: pinocytosis and phagocytosis^35, 36^. Pinocytosis can be further divided into two subcategories: macropinocytosis and micropinocytosis. Particles and solute macromolecules with diameters greater than 200 nm are non-selectively taken up via phagocytosis/macropinocytosis^37^, whereas smaller particles are internalized through micropinocytosis (clathrin-mediated, caveolae/lipid raft-mediated, and clathrin/caveolae-independent) in all cell types^38^. In our study, the hydrodynamic size of P-GNPs was 80–90 nm; therefore, we expected them to enter cells predominantly through micropinocytosis. Endocytic inhibitors were used to evaluate the involvement of different endocytic pathways in the uptake of these three types of gold nanoparticles. As shown in Fig. 6D, P-GNTs were internalized though clathrin-mediated pathways and P-GNRs were dependent on caveolae- and clathrin-mediated pathways. As reported in previous studies, clathrin-mediated endocytosis could transmit the nanoparticles through the endosomal pathway, in which a portion of the nanoparticles could be returned to the extracellular space by exocytosis. On the contrary, caveolae-mediated endocytosis passes either through the caveosome pathway, in which a portion of the nanoparticles would be removed from the endoplasmic reticulum/Golgi body, or through the endosomal pathway, in which the nanoparticles could be exocytosed from the endosome^39^. In other words, there are potentially multiple paths for exocytosis in the caveolae-mediated pathway, which may result in an apparently lower efficiency of cellular uptake of P-GNRs. Furthermore, the uptake of P-GNTs was strongly dependent on a dynamin pathway; however, P-GNRs compared to P-GNTs showed only a small usage of this pathway. Dynamin, the monomeric guanosine triphosphatase (GTPase), has been proposed to function in endocytosis^40^. Dynamin polymerizes to form rings and spirals around the necks of the pits in the fission of clathrin-coated vesicles during endocytosis^41^. GTPases are essential for the formation of vesicles and facilitate rapid vesicle retrieval, which could accelerate the trafficking of nanoparticles^42^. The internalization of P-GNTs, which strongly depended on the dynamin pathway, was much more efficient than that of P-GNRs. Support for this explanation may be found in previous study^43^. Andar *et al*. prepared liposomes to evaluate the effect of nanoparticle size on cellular uptake mechanisms and found that liposome uptake increased with a decrease in diameter and that the more efficient uptake of smaller diameter liposomes depended on their use of the dynamin pathway, which was not as utilized by the larger liposomes. Last but not the least, the activation of cytoskeletal arrangement is strongly related to the cellular uptake of P-GNTs which might be another reason for the highest uptake.

In the previous study, it was found that the different membrane bending energies during endocytosis were predominantly responsible for the nanoparticle shape effect^25^. GNSs appear to be more irregular than GNRs and GNTs, with multiple branches of different lengths (Fig. 2G–I). Therefore, the star-like nanoparticles might have to overcome a higher membrane bending energy barrier. Alternatively, before being coated with mPEG, GNSs stabilized in HEPES solution were neutrally charged, while GNRs and GNTs were highly positively charged due to the presence of the stabilizing agents CTAB and CTAC. As stated before, the PEG coating neutralized the zeta potential. It is conceivable that the small amounts of residual CTAB and CTAC on the surfaces of P-GNRs and P-GNTs might increase the affinity of the nanoparticles toward the cell membrane, which has an overall negative charge, leading to higher uptake of nanoparticles. The pure neutral charges of P-GNSs could be associated with lower affinity for the cell surface and decreased cellular uptake^44^.

In summary, Our results demonstrated that gold nanotriangles exhibited the greatest cellular uptake by RAW264.7, followed by gold nanorods and gold nanostars. We also investigated the possible mechanisms of cellular uptake. Gold nanoparticle uptake was induced via various different endocytosis mechanisms, dependent on the shape. All three shapes utilized the clathrin-mediated endocytic pathway. Gold nanorod uptake was also dependent on caveolae/lipid raft-mediated endocytosis and gold nanotriangle uptake was strongly associated with cytoskeletal rearrangement, as well as the dynamin pathway. Nanoparticle shape obviously governed the endocytosis pathways that induced the different uptake trends. We speculate that gold nanostars that have multiple branches of different lengths might have to overcome a higher membrane bending energy barrier, leading to their lower cellular uptake^25, 45–47^. Further studies should be performed to examine this theory. We have provided evidence about the importance of shape in nanoparticle-cell interactions, and our findings can be used to guide the development of GNPs for drug delivery.

## Methods

### Preparation of three types of GNPs

GNSs were synthesized through a seedless, surfactantless, and high-yield protocol modified from a method reported by Xie and coworkers^48^. A 100 mM stock solution of HEPES was prepared with deionized water, and the pH was adjusted to 7.4 ± 0.1 at 25 °C by adding 1 M NaOH solution. Then, 10 mL of 100 mM HEPES (pH 7.4) was mixed with 15 mL deionized water, and 250 μL of 25 mM chloroauric acid tetrahydrate (HAuCl_4_·4H_2_O) solution was added. After 1 h, the GNSs were purified by centrifugation at 10,000 rpm for 10 min. The precipitates were redispersed in 4 mL deionized water.

GNRs were prepared by a seed-mediated approach according to the method previously reported^49, 50^. With gentle mixing, 100 μL of 25 mM HAuCl_4_·4H_2_O was added to 7.5 mL of 0.1 M CTAB to prepared the seed solution. After 2 min, 600 μL of 10 mM sodium borohydride (NaBH_4_) solution, which had been freshly prepared and kept ice-cold, was added. The color of the seed solution changed to brownish yellow and the solution was kept undisturbed at room temperature for 2 h prior to use. Then, the growth solution was prepared. 100 mL of 0.1 M CTAB, 2.04 mL of 25 mM HAuCl_4_, 2 mL of 0.5 M H_2_SO_4_, 0.9 mL of 0.01 M AgNO_3_ and 0.8 mL of 0.1 M L-ascorbic acid were added in that order, one by one, to a flask, followed by vigorous stirring. Finally, 240 μL of seed solution was added. After 30 s of gentle mixing, the reaction mixture was left undisturbed for 12 h. Then, the GNRs were centrifuged twice at 12,000 rpm for 10 min to purify them. The precipitates were redispersed in 8 mL deionized water.

GNTs were also synthesized by a seed-mediated method described in the previous study^51, 52^. First, gold seed particles were prepared: 50 μL of a 25 mM HAuCl_4_ solution was added to 4.7 mL of 0.1 M CTAC solution; under vigorous stirring, 300 μL of 10 mM NaBH_4_ solution, which had been freshly prepared and kept ice-cold, was then added. The seed solution was kept undisturbed at 25 °C prior to use. Then, we prepared the following two growth solutions: (1) 1.6 mL of a 0.1 M CTAC solution was added to 8 mL of Milli-Q water, followed by 80 μL of 25 mM HAuCl_4_ solution and 15 μL of a 0.01 M NaI solution; (2) 1 mL of a 25 mM HAuCl_4_ solution was added to 40 mL of 0.05 M CTAC, followed by 300 μL of a 0.01 M NaI solution. Before proceeding, 0.5 mL of the initial seed solution was diluted 10× into 5 mL in a 0.1 M CTAC solution. Subsequently, 40 and 400 μL of 0.1 M L-ascorbic acid solution were added to solutions 1 and 2, respectively, and both solutions were manually stirred until they became completely transparent. Finally, 200 μL of diluted seed solution was added to solution 1 and immediately 3.2 mL of this solution was added to solution 2 with gentle mixing for few seconds. The GNTs dispersion was left undisturbed at 25 °C for 2 h. The GNT dispersion was centrifuged at 12,000 rpm for 10 min to purify the particles. Then, the precipitates were redispersed in 4 mL deionized water, followed by the addition of 2 mL 25% (wt/vol) CTAC solution and flocculation of the GNTs was completed overnight. The supernatant was then removed, and the precipitated particles were redispersed in 8 mL deionized water.

### Coating of gold nanoparticles with methylpolyethylene glycol

The method of synthesizing P-GNPs was modified from an approach reported by Han and coworkers^53^. First, 200 mg of mPEG was added into 4 mL of the prepared solutions of GNPs, followed by the addition of 32 mL of Milli-Q water. The mixed solutions were stirred at room temperature (25 °C), and incubated for 12 h to generate P-GNPs. P-GNPs were collected by centrifugation (10,000 rpm, 5 min) two times. The pellets were resuspended in deionized water. After characterization, the solutions of P-GNPs were filtered using a 0.22-μm syringe filter and stored at 4 °C. The concentrations of P-GNPs were measured by ICP-AES.

### Characterization of GNPs and P-GNPs

The ultraviolet-visible (UV-Vis) absorption spectra of prepared GNPs were confirmed by a UV-1601PC spectrophotometer. A Tecnai G2 F20 S-TWIN TEM (FEI Co., Oregon, USA) at an accelerating voltage of 120 kV was used to obtain transmission electron microscopy (TEM) micrographs. A Zetasizer Nano-ZS from Malvern Instruments (Zetasizer Nano ZSP, Malvern, England) was used to determine the particle sizes and zeta potentials. 3D structure of GNPs were obtained by atomic force microscopy (AFM) performed in tapping mode with a Shimadzu SPM-9700.

### Cell culture and CCK8 assay

RAW 264.7 cells (ATCC) were purchased from Central South University Cell Bank (Changsha, China). Cells were incubated at 37 °C (5% CO_2_) in Dulbecco’s Modified Eagle’s Medium (DMEM) supplemented with 10% (vol/vol) fetal bovine serum and 1% penicillin–streptomycin^54–56^. The CCK-8 assay was carried out to investigate cell viability. Briefly, RAW264.7 cells were seeded in a 96-well plate with 100 μL fresh growth medium at a density of 5000 cells per well and cultured overnight. On the next day, the old medium was removed and 100 μL fresh medium with serial concentrations (2.5, 5, 10, 20, 40 μg/mL) of P-GNPs was added into each well. After incubating for 24 h, the cells were washed thrice with phosphate buffered saline (PBS) and the cell viability was measured by a CCK-8 assay.

### Evaluation of Cellular Uptake of P-GNPs

RAW264.7 cells were plated in 90 × 20 mm Petri dishes at a density of 5.0 × 10^6^ cells/well. After 24 h, growth medium was removed and fresh serum free medium containing 20 μg/mL P-GNPs was added. The RAW264.7 cells were incubated for 24 h at 37 °C. Finally, the cells were washed thrice with PBS, fixed, and sectioned. Each section was placed onto a copper grid and imaged by TEM.

The quantitative evaluation of cellular uptake of P-GNPs was performed according to the method described by Her and coworkers^57^. RAW264.7 cells were plated in 6-well plates at a density of 10^6^ cells/well. On the next day, the growth medium was replaced by fresh serum-free medium containing 20 μg/mL of P-GNPs. At each time point (4, 8, and 24 h), medium was removed, cells were washed three times with PBS, and then the cells were harvested with trypsin. Cells were counted and then centrifuged at 1,000 rpm for 5 min. Supernatant was removed, and the pellet was digested with 0.5 mL of fresh aqua regia for 10 min and then diluted to a total volume of 5 mL with Milli-Q water. The concentrations of internalized gold were measured by ICP-AES, and were reported as the concentration of gold (pg) per cell.

### Inhibition studies of the endocytosis of P-GNPs

RAW264.7 cells were seeded in 6-well plates at a density of 10^6^ cells/well. After a 24 h incubation, cells were washed twice with PBS and preincubated for 1 h with the following endocytic inhibitors in serum-free medium at 37 °C: methyl-β-cyclodextrin (10 mM), Dynasore (80 μM), sucrose (450 mM), and cytochalasin (10 μg/mL). We used the concentrations of the inhibitors that were previously reported^58^. We have tested that these concentrations of the inhibitors had no obvious cytotoxicity. After 1 h, fresh medium containing the inhibitors and P-GNPs (20 μg/mL) was added after the original medium was removed, and incubation continued for another 8 h at 37 °C. Cells treated with only P-GNPs were regarded as a positive control and untreated cells were regarded as a negative control. After incubation, cells were washed thrice with PBS and harvested with trypsin, and then centrifuged at 1,000 rpm for 5 min. The pellets were digested with 0.5 mL of fresh aqua regia for 10 min and then diluted with 4.5 mL of Milli-Q water. The amounts of intracellular gold were determined by ICP-AES analysis.

Inhibition efficiency (%) was calculated by the following equation: Inhibition (%) = (Amount of P-GNP taken up in the presence of inhibitors/Amount of P-GNP taken up in the absence of inhibitors) × 100%.
